# Analysis of Diagnosis and Treatment of Active Lupus Enteritis Accompanied by Intestinal Pseudo‐Obstruction as the Initial Manifestation: A Case Report and Literature Review

**DOI:** 10.1155/crgm/3428538

**Published:** 2026-07-28

**Authors:** Wenhan Zhuang, Meijun Ji, Pengfei Xu, Lijuan Wang, Jiyong Jing, Wensheng Pan, Chenjing Zhang

**Affiliations:** ^1^ The Second School of Clinical Medicine, Hangzhou Normal University, Hangzhou, Zhejiang, China, hznu.edu.cn; ^2^ Department of Gastroenterology, Zhejiang Provincial People’s Hospital, Affiliated People’s Hospital, Hangzhou Medical College, No. 158 Shangtang Road, Hangzhou, Zhejiang, China, hznu.edu.cn; ^3^ Department of Rheumatology and Immunology, Zhejiang Provincial People’s Hospital, Affiliated People’s Hospital, Hangzhou Medical College, No. 158 Shangtang Road, Hangzhou, Zhejiang, China, hznu.edu.cn; ^4^ Department of Medical Education and Simulation Center, Zhejiang Provincial People’s Hospital, Affiliated People’s Hospital, Hangzhou Medical College, Hangzhou 310014, Zhejiang, China, hznu.edu.cn

**Keywords:** intestinal pseudo-obstruction, lupus enteritis, systemic lupus erythematosus

## Abstract

**Background:**

Intestinal pseudo‐obstruction (IPO) is a rare initial manifestation of systemic lupus erythematosus (SLE), often misdiagnosed as mechanical obstruction or malignant tumor, leading to unnecessary intervention measures and poor prognosis. Case Report: We report a 46‐year‐old female patient presenting with recurrent abdominal pain and IPO, ultimately diagnosed with SLE through multidisciplinary assessment. The patient exhibited positive antinuclear antibodies (ANAs) (+↑), homogeneous karyotype (+++ 1:1000↑), low complementemia, and lupus nephritis, meeting the diagnostic criteria for SLE. Abdominal CT showed small intestine and colon wall edema with characteristic “target sign” and mesenteric vascular congestion (“comb‐like sign”). Initial treatment included pulse therapy with methylprednisolone (240 mg/day) combined with cyclophosphamide and hydroxychloroquine, with symptom relief, but recurrence occurred multiple times during the tapering period.

**Literature Review:**

Analysis of 54 cases of SLE‐related IPO (1997‐2025) indicated:demographic characteristics: 92.6% were female, and the median age at diagnosis was 31 years. Clinical features: 81.3% of patients presented with abdominal pain; 93.0% involved the small intestine. Imaging examinations: intestinal wall edema (“target sign”) and mesenteric vascular hyperplasia (“comb‐like sign”); these signs indicate SLE‐related small bowel involvement. Immunological examinations: the positive rate of ANAs (ANA) was 75.9%, the positive rate of anti–double‐stranded DNA antibody (anti‐dsDNA) was 61.1%, and the positive rate of anti‐SSA antibody was 42.5%. Prognosis: Despite immunosuppressive treatment, the recurrence rate was 25%; the mortality rate was 7.5%.

**Conclusion:**

For patients with recurrent IPO accompanied by low albuminemia, elevated D‐dimer, and positive autoantibodies, autoimmune etiology must be considered. Early identification and immunosuppressive treatment are crucial to avoiding unnecessary surgery.

## 1. Introduction

Intestinal pseudo‐obstruction (IPO) is characterized by intestinal dilation without mechanical obstruction, presenting with pseudo‐obstruction symptoms. X‐ray or CT imaging typically shows IPO with intestinal dilation. Abdominal distension is the main clinical feature, and it may also be accompanied by nonspecific symptoms such as nausea, vomiting, early satiety, constipation, diarrhea, and generalized abdominal pain [1]. Currently, the treatment strategy for IPO mainly focuses on symptom management, including decompression of dilated bowel and prokinetic intervention. Thorough investigation and resolution of underlying causes are crucial, and targeted treatment addressing the root cause is key to achieving fundamental clinical improvement and optimizing treatment outcomes.

Systemic lupus erythematosus (SLE) is a systemic inflammatory disease mediated by immune dysregulation, characterized by multiorgan involvement, including the skin and mucous membranes, musculoskeletal system, hematological system, and renal system. SLE‐related intestinal lesions are mainly classified into three types: mesenteric vasculitis, IPO, and protein‐losing enteropathy (PLE). In current case reports, there is both overlap and distinction among the three. Sjögren’s syndrome (SjS) is characterized by lymphocyte proliferation and progressive dysfunction of exocrine glands, mainly affecting the lacrimal and salivary glands, leading to typical symptoms such as dry eyes and dry mouth [2]. Epidemiological studies have shown that the prevalence of SjS in SLE patients ranges from 14% to 17.8% [3]. IPO is often misdiagnosed due to its nonspecific symptoms and may be confused with mechanical intestinal obstruction or malignancy, with a reported misdiagnosis rate of approximately 40% [4]. Such diagnostic errors can lead to delayed appropriate treatment and unnecessary interventions, significantly compromising patient prognosis.

In this study, a patient initially presented to the emergency department and surgery with IPO. Despite symptomatic treatment, the symptoms persisted. The patient was eventually diagnosed with SjS/SLE overlap syndrome and referred to the rheumatology department for targeted treatment, with significant improvement in the condition. Early identification of IPO is of great clinical significance in avoiding unnecessary surgery and improving prognosis. Currently, there is no systematic elaboration on severe intestinal motility disorders in the context of autoimmune diseases. By integrating the present instructive case and previously published literature, we further characterized the clinical, imaging, therapeutic, and prognostic profiles of active lupus enteritis (LE) accompanied by IPO, aiming to provide comprehensive evidence for the early diagnosis and individualized management of this easily overlooked but clinically severe autoimmune gastrointestinal manifestation.

## 2. Case Presentation

**FIGURE 1:**
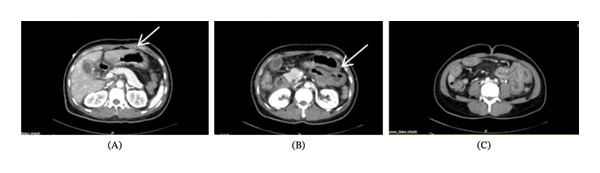
Marked focal wall thickening of the small intestine in the right lower quadrant with associated luminal narrowing, suggestive of a space‐occupying lesion.

A 46‐year‐old female patient presented to the emergency department of our institution on May 16, 2024, reporting persistent abdominal pain lasting more than one month. Her chief complaint was generalized, cramping, persistent, and progressively worsening abdominal pain, accompanied by nausea, recurrent postprandial vomiting with gastric contents, and complete cessation of both defecation and flatus. Contrast‐enhanced abdominal computed tomography (CT; Figure 1) demonstrated marked focal small bowel wall thickening with luminal stenosis in the right lower quadrant, raising suspicion for an underlying space‐occupying lesion requiring further evaluation. Specifically, the small bowel demonstrated a multilayered hyperdense and hypodense alternating wall appearance forming a classic target sign. In addition, increased mesenteric vascular dilatation and hypervascularity presented as a comb‐like sign, suggestive of mesenteric inflammatory hyperperfusion. Additional imaging findings included wall thickening and edema involving the 2nd to 4th segments of the small intestine (extending from the jejunum to the proximal ileum), concurrent ascites, and bilateral pleural effusions. The patient was diagnosed with IPO and developed fever (38.2°C) during hospitalization. Laboratory workup revealed mild anemia, elevated inflammatory biomarkers, abnormal arterial blood gas indices, and significantly increased D‐dimer levels. Medical management included prophylactic anticoagulation for thrombus prevention, nil per os status with gastrointestinal decompression, and abdominal paracentesis with drainage. Gastrointestinal decompression was initiated immediately after admission due to severe intestinal distension and vomiting to reduce intraluminal intestinal pressure, protect intestinal mucosal perfusion, and relieve obstructive symptoms. Biochemical analysis of the ascitic fluid yielded unremarkable results. Following 12 days of targeted supportive and symptomatic treatment, the patient’s clinical symptoms improved, and she was discharged in a stable condition.

**FIGURE 2:**
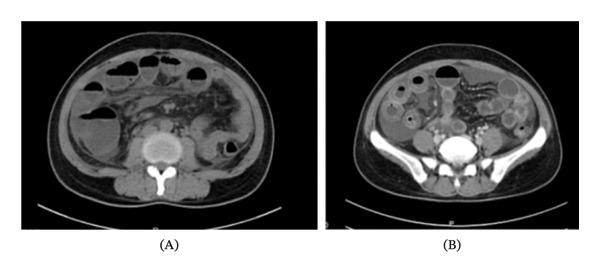
Wall thickening of the ascending and transverse colon, with edematous wall thickening and exudative changes observed in portions of the small bowel, suggestive of inflammatory pathology, and dilated bowel loops with air‐fluid levels, consistent with incomplete intestinal pseudo‐obstruction.

One month after the initial hospitalization (June 2024), the patient re‐presented to the emergency department with recurrent paroxysmal abdominal pain accompanied by nausea and vomiting. Contrast‐enhanced abdominopelvic CT with multiplanar reconstruction of the small intestine (Figure 2) identified wall thickening of the ascending and transverse colon, segmental small bowel edema with exudative changes indicative of inflammatory pathology, intestinal dilatation with air‐fluid levels consistent with partial obstruction, abdominopelvic effusion, and enlargement of retroperitoneal and mesenteric lymph nodes. Incidental findings included dilatation of the right hepatic bile duct, an accessory spleen, and mild dilatation of the right ureter. Segmental small bowel edema was accompanied by recurrent target sign changes, and mesenteric vascular proliferation and hyperemia again presented as a comb sign. Upon admission to the gastroenterology department, laboratory investigations revealed a serum albumin level of 29.4 g/L (reference range decreased), marked proteinuria (+++), microalbuminuria greater than 300 mg/L (+++), and a urine albumin/creatinine ratio (ACR) exceeding 30 mg/mmol (++), along with a markedly elevated plasma D‐dimer level of 10,810.0 μg/L (reference range increased). Autoimmune serological testing was positive for antinuclear antibody (ANA; +) with a homogeneous pattern at a titer of 1:1000 (+++), positive anti–extractable nuclear antigen (anti‐ENA) antibodies, and elevated levels of anti‐SSA (60KD; 93 U/mL, +++), anti‐Ro52 (105 U/mL, +++), and anti‐SSB (97 U/mL, +++) antibodies; antiphospholipid antibodies were negative. Endoscopic evaluation confirmed chronic nonatrophic gastritis, carditis, duodenal edema, and terminal ileitis. Rheumatologic assessment with salivary gland ultrasound showed bilateral atrophy of the parotid and submandibular glands, cervical lymphadenopathy, and a left thyroid nodule. Labial salivary gland biopsy verified focal lymphocytic infiltration (> 50 lymphocytes per focus), consistent with a diagnosis of SjS. Positron emission tomography/CT (PET/CT) demonstrated mesenteric panniculitis with fluorodeoxyglucose (FDG)–avid lymph nodes, reduced small bowel wall thickening compared to prior imaging, decreased pelvic effusion, FDG uptake in the gastric antrum consistent with inflammatory changes, chronic sinusitis, and salivary gland calcifications. During the initial emergency hospitalization, a differential diagnosis was conducted for the patient’s IPO‐like symptoms. Mechanical intestinal obstruction was excluded given the absence of obstructive masses and the presence of diffuse bowel inflammatory edema on CT. Infectious enteritis and toxic bowel paralysis were ruled out based on negative infectious parameters, sterile ascitic fluid, and normal stool findings. Although primary IPO was considered, it was less favored because the acute presentation was accompanied by serous effusion, elevated D‐dimer, and systemic inflammation, indicating a secondary systemic etiology rather than primary intestinal dysmotility.

After fluid resuscitation and gastrointestinal decompression, the patient underwent surgical resection of the thyroid nodule due to suspicious histologic features, with a scheduled rheumatology follow‐up appointment in 2 weeks to formulate a treatment plan for SjS.

The patient presented to the rheumatology department on July 21, 2024 (2 weeks after thyroidectomy) with recurrent nausea and vomiting. Further targeted history taking elicited a 6‐year history of xerostomia, xerophthalmia, alopecia, occasional photosensitivity, and intermittent arthralgia involving the small joints of the hands with associated morning stiffness. Repeat laboratory testing showed decreased serum complement C3 (0.79 g/L, reference range decreased), nephrotic‐range proteinuria (urine protein/creatinine ratio 1.94 g/g), and severe microalbuminuria. Contrast‐enhanced abdominal CT revealed focal wall thickening of the stomach, duodenum, and ascending colon, suggestive of inflammatory mucosal changes (endoscopic reevaluation recommended). Compared to imaging studies from June 21, 2024, partial resolution of IPO and reduced abdominopelvic effusion and exudation were noted. Comprehensive diagnostic workup confirmed the following diagnoses: (1) SLE; (1.1) lupus‐related gastrointestinal involvement; (1.2) partial IPO; and (1.3) lupus nephritis. This patient fulfilled the 2019 EULAR/ACR SLE classification criteria (see Table 1). The patient received 5 days of intravenous methylprednisolone pulse therapy (240 mg once daily, July 24 to 29, 2024) but reported persistent abdominal distension with no symptomatic improvement. A noncontrast abdominopelvic CT performed on July 30, 2024, showed reduced wall thickening of the stomach and duodenum compared to July 22, 2024, but worsened edema of the right colonic wall, increased abdominopelvic fluid and exudate, irregular urinary bladder wall contour, and multiple enlarged retroperitoneal and mesenteric lymph nodes. Colonoscopy confirmed colitis secondary to underlying SLE. The patient still suffered from persistent abdominal distension, indicating an inadequate therapeutic response to the initial high‐dose pulse regimen. Corticosteroid therapy was subsequently adjusted: intravenous methylprednisolone 120 mg once daily (July 30–August 1, 2024), followed by 40 mg once daily (August 2–8, 2024). We excluded patients with severe myelosuppression, significant hepatic/renal dysfunction, active severe infection, pregnancy, and a history of drug allergy to alkylating agent therapy. This was supplemented with intravenous cyclophosphamide (CTX) 0.4 g (after contraindication screening) and oral hydroxychloroquine 0.1 g three times daily for immunomodulatory therapy. Maintenance immunosuppressive therapy was switched to oral methylprednisolone 36 mg once daily, combined with adjuvant antibiotics, proton pump inhibitors, anticoagulants, calcium and potassium supplementation, and albumin replacement therapy. The patient’s clinical symptoms resolved gradually, and she was discharged home.

**TABLE 1:** Assessment of the patient against the 2019 EULAR/ACR SLE classification criteria.

Domain	Item	Patient’s manifestation	Score
Mandatory criterion	ANA (HEp‐2 cell assay)	ANA (HEp‐2 cell assay)	Entry

Clinical domains	General symptoms	Unexplained fever (38.2°C)	2
	Cutaneous/mucosal manifestations	Alopecia, intermittent photosensitivity	2
	Musculoskeletal involvement	Intermittent arthralgia of small joints with morning stiffness	6
	Serositis	Ascites, bilateral pleural effusions	5
	Renal involvement	Nephrotic‐range proteinuria	4
	Hematological abnormalities	Mild anemia	3

Immunological domains	Complement reduction	Decreased C3	3
	SLE‐specific autoantibodies	Negative	0
	Antiphospholipid antibodies	Negative	0

Total score			25

Following discharge (August 2024 to present), the patient remained asymptomatic with no recurrence of abdominal pain, distension, chills, or fever. Long‐term maintenance therapy consisted of monthly intravenous CTX 0.8 g, continued oral hydroxychloroquine 0.1 g three times daily, and oral methylprednisolone 32 mg once daily to control the underlying autoimmune disease. In October 2024, the patient experienced a 5‐h episode of acute abdominal pain, which resolved following treatment with intravenous isepamicin 0.4 g. Hydroxychloroquine was discontinued in March 2025 due to recurrent dizzy spells. Neurological assessment excluded cerebrovascular disease, anemia, and electrolyte disturbance, and the dizziness was considered a probable adverse reaction to hydroxychloroquine. After thorough evaluation, hydroxychloroquine was discontinued. Considering the patient’s stable disease state and intolerance to this agent, we did not initiate alternative antimalarial drugs. Ongoing clinical monitoring under regular rheumatology follow‐up has demonstrated stable disease status to date.

## 3. Methods and Results

### 3.1. Methods

A structured literature review was performed, with a total of 37 English‐language articles ultimately included for analysis. These studies encompassed 54 individual patients who fulfilled the American College of Rheumatology (ACR) revised classification criteria for SLE and in whom IPO was documented as a clinical manifestation attributable to underlying SLE pathogenesis. Disease activity was quantitatively assessed using the Systemic Lupus Erythematosus Disease Activity Index (SLEDAI), the most extensively validated and widely utilized scoring tool for SLE disease activity monitoring. Literature searches were conducted in the PubMed/MEDLINE database spanning the period from January 1997 to March 2025, employing the following Medical Subject Headings (MeSH) terms and keyword combinations: intestinal pseudo‐obstruction and systemic lupus erythematosus; supplementary searches using the abbreviations “IPO” and “SLE” were also performed to enhance retrieval sensitivity.

To ensure comprehensive literature capture, reference lists of all included full‐text articles and previously published narrative reviews on related topics were manually screened to identify eligible studies potentially missed by the primary electronic search strategy. Detailed baseline characteristics, clinical data, and outcomes of all included patients are summarized in Supporting Tables 1 and 2.

Inclusion criteria: Original clinical studies, including cohort studies, case series, and informative case reports, were eligible if they enrolled patients diagnosed with SLE (2019 EULAR/ACR or 2012 SLICC criteria) and clinically/radiologically confirmed IPO. Eligible articles had complete demographic, clinical, imaging, treatment, and follow‐up data, were published in English or Chinese, and had accessible full texts. A standardized self‐designed data extraction form was used for data collection. Two experienced researchers independently extracted data from all 37 eligible articles. The extracted information included general patient information, clinical symptoms, imaging features, laboratory indicators, treatment strategies, and prognosis data.

Exclusion criteria: Exclusions included reviews, letters, editorials, conference abstracts, and basic experimental studies without original clinical data. Articles with duplicate or overlapping patient data, insufficient extractable clinical information, or cases of primary IPO without underlying SLE were also excluded.

### 3.2. Results

#### 3.2.1. Baseline Clinical Characteristics

The final study cohort comprised 54 patients, with a marked female predominance (male‐to‐female ratio = 4:50). IPO represented the initial clinical manifestation of SLE in 29 patients, accounting for 53.7% of the entire cohort. The mean age at SLE diagnosis was 28.2 years, while the mean age at IPO onset was 31.0 years. Following initial immunosuppressive treatment, gastrointestinal symptom recurrence was documented in 25% of patients, and 4 patients (7.4%) succumbed to progressive disease‐related complications.

The most prevalent clinical manifestations included abdominal pain (81.3%) and vomiting (72.9%). Small intestinal involvement was the predominant gastrointestinal phenotype, affecting 93% of patients, with 11 cases demonstrating diffuse intestinal dilatation and edema. Characteristic contrast‐enhanced CT findings consistent with lupus‐related enteropathy and vasculopathy included the following: (1) the “target sign,” defined as concentric small bowel wall edema and circumferential thickening and (2) the “comb sign,” indicative of engorged and prominent mesenteric vasculature.

The duration of prehospitalization symptom onset ranged from 3 days to 3 years. Notably, 75% of patients achieved a favorable clinical response to systemic corticosteroid and/or immunosuppressive therapy, with clinical improvement observed within a timeframe of 2 days–3 months of treatment initiation.

#### 3.2.2. Urinary Tract Involvement

Concurrent urinary tract manifestations were highly prevalent in this cohort: Ureteral dilatation and hydronephrosis were identified in 42 patients (77.8%), while bladder wall thickening was present in 6 cases (11.1%).

#### 3.2.3. Immunological and Laboratory Features

Autoantibody seropositivity rates among the cohort were as follows: ANA, 75.9%; anti–double‐stranded DNA (anti‐dsDNA) antibody, 61.1%; and anti‐Ro (SSA) antibody, 42.5%. Hypokalemia (serum potassium concentration < 3.5 mmol/L) was detected in 8 of 12 tested patients (66.7%).

The mean SLEDAI score for the cohort was 15 points (range: 12–22), confirming moderate to high SLE disease activity across the study population. Reduced serum complement levels, a hallmark of active SLE, were observed in 36 patients (66.7%).

#### 3.2.4. Therapeutic Interventions and Clinical Management

CTX emerged as the most frequently administered first‐line immunosuppressive agent, utilized in 62.9% of patients. Notably, 14.8% of patients underwent surgical intervention due to suspected mechanical bowel obstruction prior to definitive diagnosis of IPO, suggesting potential gaps in early diagnostic recognition and conservative management strategies.

Combination therapy with CTX and systemic glucocorticoids was prescribed in 27.8% of cases, consistent with current standard‐of‐care regimens for severe, active SLE with life‐threatening organ involvement.

Conversely, the use of cyclosporine was exceedingly rare (3.7%), indicating a limited clinical role for this agent in the management of SLE‐related IPO.

## 4. Discussion

This case report describes a 46‐year‐old female patient who initially presented to the emergency department with clinical and radiological features suggestive of acute intestinal obstruction, without prior diagnosis of a systemic autoimmune disorder. Following multidisciplinary evaluation and collaborative discussion involving gastroenterology, rheumatology, and radiology teams, the patient was ultimately diagnosed with SLE–IPO. Notably, the patient achieved rapid and sustained clinical remission following administration of systemic glucocorticoids and sequential immunosuppressive therapy, consistent with the therapeutic response pattern observed in cases of severe active lupus‐related gastrointestinal involvement.

LE, a major gastrointestinal manifestation of active SLE, is defined by the British Isles Lupus Assessment Group (BILAG) as small intestinal vasculitis or inflammatory enteropathy confirmed by cross‐sectional imaging or histopathological biopsy, following rigorous exclusion of alternative etiologies including infectious enterocolitis, mechanical obstruction, thrombotic disease, and primary inflammatory bowel disease [5]. Recent clinical evidence has demonstrated a significant positive correlation between elevated serum D‐dimer levels and the development of active LE, with hyper‐D‐dimeremia in this context primarily attributed to SLE‐driven systemic inflammation and endothelial activation, rather than overt thrombotic events [6]. Accordingly, prophylactic anticoagulation was administered in this patient to mitigate thromboembolic risk, a standard adjunctive measure in patients with active SLE and severe intestinal vasculopathy.

In the present case, IPO occurred as the initial clinical presentation of underlying SLE, representing an uncommon and frequently misdiagnosed phenotype of lupus gastrointestinal involvement. Integrated analysis of clinical, laboratory, and radiological findings confirmed the diagnosis of LE, with the core pathogenesis centered on SLE‐induced small intestinal microvasculitis and immune‐mediated transmural intestinal wall inflammation. While the precise molecular mechanisms underlying lupus‐related vasculitis remain incompletely elucidated, the complex crosstalk between vascular endothelial injury, inflammatory cell infiltration, proinflammatory cytokine release, circulating autoantibodies, and vascular immune complex deposition is widely recognized as a central pathogenic driver [7]. LE is classified as a secondary autoimmune vasculitis, which may manifest with acute or subacute clinical features, and its inflammatory cascade is primarily triggered by complement activation and immune complex deposition within the walls of small mesenteric vessels. Lupus‐related autoimmune mesenteric vasculopathy induces bowel wall edema and peristaltic dysfunction, leading to secondary IPO features such as abdominal distension, vomiting, and obstipation. Hence, LE is the primary underlying disorder, with IPO‐like findings as a secondary clinical phenotype.

During the third hospitalization, repeated abdominal imaging revealed prominent mesenteric lymphadenopathy, progressive small intestinal edema, and recurrent ascites, prompting clinical suspicion of concurrent PLE [8]. The patient exhibited severe hypoalbuminemia (29.4 g/L), which could not be fully attributed to nephrotic‐range proteinuria secondary to lupus nephritis alone, despite documented renal protein loss. We hypothesize that the combination of renal protein wasting, severe systemic inflammatory burden, and intestinal protein leakage due to vasculitis‐mediated mucosal injury collectively contributed to profound hypoalbuminemia in this case. Given that the degree of proteinuria was insufficient to account for the marked reduction in serum albumin, PLE was considered a critical contributing factor to hypoalbuminemia in this patient. Definitive evaluation for PLE typically involves assessment of α1‐antitrypsin clearance or radionuclide scintigraphy with 99mTc‐labeled albumin, which were not performed in this case and represent a diagnostic limitation. Previous studies have validated a highly specific diagnostic combination for PLE: serum albumin < 22 g/L plus 24‐h urinary protein excretion < 0.8 g, yielding a sensitivity of 0.818 and specificity of 0.989 [9].

This case and accompanying narrative review also address a key clinical conundrum: severe gastrointestinal dysmotility in the setting of systemic autoimmune diseases, particularly LE. Chronic IPO (CIPO) is defined by persistent clinical and radiological signs of intestinal obstruction (including dilatation and air‐fluid levels on cross‐sectional imaging) in the absence of mechanical occlusion, with symptom duration of at least 6 months required to confirm chronicity [10]. While CIPO and primary intestinal dysmotility disorders represent distinct clinicopathological subtypes with divergent prognostic and therapeutic implications, they are often clinically grouped as a single phenotypic entity. CIPO constitutes the most severe form of chronic intestinal dysmotility, characterized by impaired small intestinal peristalsis and classical obstructive radiological signs without structural or mechanical obstruction. Per the classification proposed by Alcala et al., this case represents secondary CIPO associated with systemic autoimmune disease, for which small intestinal manometry is indicated for objective functional assessment [11]. A core diagnostic step remains rigorous exclusion of mechanical obstruction or structural lesions, followed by objective evaluation of small intestinal motility via high‐resolution manometry (HRM). This patient presented with secondary IPO triggered by autoimmune injury. Notably, the disease duration did not reach the 6‐month threshold required for a formal diagnosis of CIPO. Therefore, this discussion focuses on the full spectrum of SLE‐related intestinal dysmotility and secondary IPO, rather than classic CIPO.

Diagnostically, this case relied heavily on cross‐sectional imaging findings and serial clinical trajectory, rather than objective functional testing of intestinal motility. Such imaging features are suggestive of small intestinal involvement secondary to systemic disease but are not pathognomonic for IPO, representing a notable limitation under current diagnostic criteria [11]. HRM has been established as superior to conventional manometry for identifying distinct patterns of intestinal dysmotility, with critical utility for diagnostic stratification, phenotypic characterization, and prognostic prediction in patients with CIPO [12]. Additionally, full‐thickness intestinal biopsy should be considered in patients with autoimmune‐related idiopathic CIPO, while genetic testing holds promise for clarifying underlying pathogenic mechanisms, defining more homogeneous patient subgroups, and facilitating early diagnosis and targeted therapeutic strategies for this rare and heterogeneous disorder.

The HRM technique described by Alcalá‐González et al. [13] is applicable to this clinical scenario. High‐resolution small bowel manometry outperforms conventional sparse‐sensor manometry for identifying subtle small intestinal dysmotility by capturing aberrant contractile propagation and phase‐specific motility abnormalities undetectable on limited‐channel recordings; this advanced testing therefore serves as a valuable diagnostic tool to differentiate primary IPO from IPO‐like secondary gut dysmotility induced by mesenteric vasculitis in LE. However, due to clinical reasons, these tests were not performed on this patient (Table 2).

**TABLE 2:** Baseline demographics and clinical profiles of 54 enrolled patients.

Demographics	Data
Gender (female), *n*/*N* (%)	50/54 (92.6%)
Age of onset of SLE, mean	28.20
Age of onset of IPO, mean	31.00
IPO recurrence, *n*/*N* (%)	12/48 (25.0%)
Survival, *n*/*N* (%)	49/53 (92.5%)
Abdominal pain, *n*/*N* (%)	39/48 (81.3%)
Abdominal distension, *n*/*N* (%)	20/48 (41.7%)
Vomiting, *n*/*N* (%)	35/48 (72.9%)
Constipation, *n*/*N* (%)	10/48 (20.8%)
Diarrhea, *n*/*N* (%)	15/48 (31.2%)
Located in the small intestine, *n*/*N* (%)	40/43 (93.0%)
CT “target sign,” *n* (edema and thickening of the bowel wall)	19
CT “Comb sign,” *n* (enhanced mesenteric vascular filling)	5

This study includes a narrative review of 54 published cases of SLE‐associated IPO from 1997 to 2025, with pooled statistical analysis confirming a striking female predominance (92.6%), consistent with established female predilection in SLE. Previous epidemiological data report an incidence of 3.8%–5.2% for IPO among patients with SLE, highlighting its status as a rare but severe complication [14]. Importantly, IPO may occur either as the initial presenting manifestation of SLE or as a late complication during the disease course. Secondary IPO associated with primary SjS is rarer, with an estimated incidence of 2.4%, and existing evidence is limited to a single Japanese survey [15].

Pooled analysis of clinical manifestations in SLE‐related IPO revealed the most frequent gastrointestinal symptoms: abdominal pain (81.3%), vomiting (72.9%), diarrhea (31.2%), constipation (20.8%), and abdominal distension (41.7%) (Table 3). Small intestinal involvement may precipitate small intestinal bacterial overgrowth (SIBO) and fat malabsorption, leading to chronic diarrhea, while colonic involvement is more commonly associated with constipation [16]. The mean SLEDAI score in this cohort was 15 points, indicative of moderate‐to‐high disease activity. Gastrointestinal involvement is widely regarded as a marker of active SLE; however, gastrointestinal symptoms are not incorporated into the standard SLEDAI scoring system, potentially leading to underestimation of gastrointestinal disease activity and treatment response [17].

**TABLE 3:** Laboratory findings, SLEDAI scores, and therapeutic regimens of 54 enrolled patients.

Characteristics	Number (%) of patients
Hypokalemia, *n*/*N* (%)	8/12 (66.7%)
ANA, *n*/*N* (%)	41/54 (75.9%)
Anti‐dsDNA, *n*/*N* (%)	33/54 (61.1%)
Anti‐SSA, *n*/*N* (%)	23/54 (42.5%)
Anti‐SSB, *n*/*N* (%)	8/54 (14.8%)
Anti‐RNP, *n*/*N* (%)	10/54 (18.5%)
Anti‐sm, *n*/*N* (%)	13/54 (24.1%)
Low C3/C4, *n*/*N* (%)	36/54 (66.7%)
SLEDAI, Mean	15.14
Hydroureteronephrosis, *n*/*N* (%)	42/54 (77.8%)
Thickening of the bladder wall, *n*/*N* (%)	6/54 (11.1%)
Pleural effusion, *n*/*N* (%)	14/54 (25.9%)
Ascites, *n*/*N* (%)	24/54 (44.4%)
Bile duct dilation, *n*	3

*Note:* Hypokalemia was defined as serum potassium level < 3.5 mmol/L.

Optimal management of SLE‐related IPO requires individualized, multidisciplinary collaborative care, with immunosuppressive therapy tailored to the severity of organ involvement. For life‐threatening or organ‐damaging gastrointestinal manifestations, high‐dose systemic glucocorticoid pulse therapy is recommended as first‐line induction therapy [18]. Notably, current European League Against Rheumatism (EULAR) and ACR treatment guidelines do not provide specific recommendations for the management of SLE‐related gastrointestinal manifestations [19, 20]. Core therapeutic components include immunomodulatory agents (primarily glucocorticoids and CTX), prokinetic medications, broad‐spectrum oral antibiotics for SIBO prophylaxis, and parenteral nutrition support in clinically unstable patients. Per the expert consensus guidelines from ERN ReCONNET–SLICC–SLEuro, the preferred induction regimen combines hydroxychloroquine with intravenous methylprednisolone, followed by oral glucocorticoid tapering (0.25–0.5 mg/kg/day) and intravenous CTX (EuroLupus protocol: 500 mg fixed dose every 2 weeks for 6 total doses), with maintenance therapy using mycophenolate mofetil (or azathioprine for patients with concurrent pancreatitis) [21]. Approximately 44% of survey respondents in consensus guidelines endorsed a less intensive regimen (hydroxychloroquine, oral glucocorticoids 0.5–1 mg/kg/day, and mycophenolate mofetil 2–3 g/day) for nonsevere LE. Hydroxychloroquine exerts therapeutic effects via inhibition of toll‐like receptor (TLR) signaling, reduction of proinflammatory cytokine release, preservation of exocrine gland function, and decreased lymphoma risk in SjS; however, this patient developed recurrent dizziness following hydroxychloroquine initiation, a known adverse effect highlighted in the 2025 ACR guidelines that necessitated drug discontinuation. In our narrative review, 27.8% of patients received combined CTX and glucocorticoid therapy, while only 3.7% were treated with cyclosporine, reflecting the limited role of calcineurin inhibitors in this clinical setting. Prompt immunosuppressive treatment is critical to prevent irreversible smooth muscle atrophy and fibrosis, which would result in permanent loss of intestinal peristaltic function. Surgical intervention may be considered in select cases, such as suspected concurrent mechanical obstruction, or acute life‐threatening IPO complications including intestinal perforation, ischemia, or necrosis [22].

## 5. Conclusion

LE should be considered early in patients presenting with obstruction‐like symptoms when the CT findings are highly suggestive. IPO is a rare but severe manifestation of SLE, presenting either as the initial disease symptom or developing during its course. This message would be particularly useful for nonrheumatologists, including physicians in emergency medicine, gastroenterology, and surgery. After excluding vascular, mechanical, and primary dysmotility causes, additional tests including small intestinal manometry and full‐thickness biopsy are recommended for patients with IPO as the initial presentation and poor response to conservative care. SLE‐related IPO is mainly induced by intestinal microvasculitis and immune‐mediated wall inflammation, while pseudo‐obstruction is a secondary functional disorder, and hypoalbuminemia (mostly from lupus nephritis) aggravates disease severity. Early high‐dose glucocorticoids combined with immunosuppressants constitute the optimal treatment, effectively relieving symptoms, preventing irreversible intestinal smooth muscle fibrosis, avoiding unnecessary surgery, and ultimately reducing patient morbidity and mortality.

## Funding

This study was funded by the Natural Science Foundation of Zhejiang Province, 2022RC106 and 2024KY662; the Medical and Health Science and Technology Project of Zhejiang Province, Z24H030011 and 2022KY077; the Department of Science and Technology of Zhejiang Province, 2025C02061.

## Consent

No written consent has been obtained from the patients as there are no patient identifiable data included in this case report.

## Conflicts of Interest

The authors declare no conflicts of interest.

## Supporting Information

Additional supporting information can be found online in the Supporting Information section.

## Supporting information


**Supporting Information** All supporting information includes two supporting tables derived from [23–54]. Supporting Table 1 summarizes demographic characteristics, core clinical manifestations, and auxiliary examination results of patients with intestinal pseudo‐obstruction. Supporting Table 2 presents laboratory test data and extraintestinal organ involvement information among the 54 enrolled subjects [23–54].

## Data Availability

All the data used in this study for descriptive statistics were obtained from the papers published by other researchers. We are unable to provide the original data upon request.
