# Supplementary material for: Analysis of Diagnosis and Treatment of Active Lupus Enteritis Accompanied by Intestinal Pseudo‐Obstruction as the Initial Manifestation: A Case Report and Literature Review
Source: Case Rep Gastrointest Med. 2026 Jul 28;2026:3428538. doi: 10.1155/crgm/3428538 (PMC13409340; doi:10.1155/crgm/3428538)
Supplement: Supplementary file 1 — Supporting Information All supporting information includes two supporting tables derived from [23–54]. Supporting Table 1 summarizes demographic characteristics, core clinical manifestations, and auxiliary examination results of patients with intestinal pseudo‐obstruction. Supporting Table 2 presents laboratory test data and extraintestinal organ involvement information among the 54 enrolled subjects [23–54]. [file CRGM-2026-3428538-s001.docx]

Supplementary Table 1

Demographics and key clinical and investigative findings in intestinal pseudo-obstruction

| publication | Age/gender | nationality | Age of onset of SLE | Weight  loss | SLEDAI | Site of  lesion | Initial treatment | Maintenance | Operation | Prognosis |
| --- | --- | --- | --- | --- | --- | --- | --- | --- | --- | --- |
| MUNYARD P et al.[23] | 15/F | Indonesian | 15 | 3kg/3w |  | S,L | MP+HCQ（+） | oral steroids | laparotomy | no recurrences |
| KAMIUCHI Ket al,[24] | 43/F | Japan | 29 |  |  | S | MP+CTX（-） | betamethasone |  | recurrences |
| GPerlemuter et al[25] | 28/F | / | 21 | 5kg |  | S | / | / |  | No recurrence |
|  | 29/F | / | 28 | 12kg |  | S | / | / | laparotomy | No recurrence |
|  | 34/F | / | 33 | 40kg |  | S | / | / |  | died |
| PA Het al[26] | 40/F | / | 38 | 32kg |  | S | Pred+HCQ+CTX（+) | Pred+CsA | laparotomy | recurrences |
| MY Mok et al[27] | 36/F | / | 36 |  |  | / | MP+5-fu | - | laparotomy | Died（ renal failure and fungal peritonitis） |
|  | 21/F | / | 21 |  |  | S | Pred | Pred+AZA | laparotomy | recurrences |
|  | 42/F | / | 42 |  |  | S | MP | MP+CTX/AZA | laparotomy | recurrences |
|  | 25/F | / | 25 |  |  | S | Pred |  | laparotomy | no recurrences |
|  | 20/F | / | 20 |  |  | S,L | MP | Pred/MP+AZA |  | recurrence |
|  | 41/M | / | 25 |  |  | S | MP | AZA |  | no recurrences |
| J.Narvaez[28] | 44/F | Spain | 35 |  |  | S,L | MP |  |  | no recurrences |
| Alexopoulou[29] | 32/F | Asian | 25 | 20kg |  | S,L | MP+CTX | Corticosteroids |  | no recurrences |
| Mao-Yuan Chen[30] | 24/F | / | 24 |  |  | caecum ,  ileum | MP | Pred | laparotomy | no recurrences |
|  | 24/F | / | 24 |  |  | / | Pred/MP+HCQ+CTX（-） |  |  | Died (infection) |
|  | 37/F | / | 36 |  |  | / | MP |  | laparotomy | Died (infection) |
|  | 20/M | / | 20 |  |  | L | MP |  |  | no recurrences |
| Yang-YuanChen[31] | 47/M | / | 47 |  |  | S(ileum) | Pred+HCQ | Pred |  | no recurrences |
| Maruoka [32] | 23/F | Japan | 15 |  |  | / | Corticosteroids+CsA（-）/CTX、AZA | tacrolimus |  | no recurrences |
| Ceccato[33] | 24/F |  | 23 |  | 11 | S | CTX |  |  | no recurrences |
|  | 21/F |  | 17 |  | 5 | S,L | MP+CTX |  |  | no recurrences |
|  | 25/F |  | 23 |  | 12 | sigmoides | MP+CTX |  |  | no recurrences |
|  | 49/F |  | 49 |  | 11 | L | MP |  |  | no recurrences |
| Frederick D[34] | 46/F | Japanese-American | 30 |  |  | S,L | MP | MP |  | recurrences |
| Leonardi,G[35] | 51/F |  | 38 |  |  | S,L | MP | MP |  | no recurrences |
| Ghannouchi Jaafoura[36] | 24/F | France | 24 |  |  | / | Pred+HCQ（-）/corticosteroids |  |  | recurrences |
| kim[37] | 20/M |  | 20 |  |  | S | MP | Steroid+AZA | laparotomy | no recurrences |
| zhang[38] | 18/F |  | 27 |  | 20 | S | MP | Pred+leflunomide |  | no recurrences |
|  | 48/F |  | 48 | 10kg | 2 | S | MP | Pred | laparotomy | no recurrences |
| Chen[14] | 24/F |  | 24 |  |  | S,L | MP(-)/MP+IVIG | MP |  | recurrence |
| Kansal,A[39] | 27/F |  | 25 |  |  | S,L | CTX | Pred+HCQ |  | no recurrences |
| Khairullah,  [40] | 42/F |  | 42 | 13kg |  | stomach ,S,L | steroids | HCQ+AZA | laparotomy | recurrences(non-compliance） |
| Garcia Lopez[41] | 27/F |  | 27 | 10kg | 27 | / | Steroids+IVIG | Pred+mycophenolate | laparotomy | no recurrences |
|  | 23/F |  | 23 | 15kg | 11 | / | MP+IVIG | Steroids+CTX | laparotomy | recurrences |
|  | 25/F |  | 25 | 8kg | 15 | / | MP | MP+CTX |  | lost |
| wang[42] | 42/F |  | 42 | 7kg |  | / | MP | Pred |  | no recurrences |
| oh[1] | 43/F |  | 43 | 4-5kg |  | S | Pred | - |  | recurrences |
| Ji,C[43] | 26/F |  | 26 |  | 10 | jejunal | MP+gamma globulin | MP | laparotomy | no recurrences |
| Yang,C[44] | 39/F |  | 39 |  |  | S,L | Pred | Pred | laparotomy | recurrence once |
| Shirleley[45] | 38/F |  | 21 |  |  | S | MP+IG | Pred | laparotomy | no recurrences |
|  | 23/F |  | 18 |  |  | S | hydrocortisone | MP/hydrocortisone |  | no recurrences |
| wang[46] | 49/F |  | 49 |  |  | S | MP+ Corticosteroid |  |  | no recurrences |
| Adler[47] | 40/F |  | 30 |  |  | S | Pred+CTX | CTX/mycophenolate mofetil |  | no recurrences |
| Zhang,j[48] | 31/F |  | 36 |  |  | S | MP+IG |  | laparotomy | died |
| Nyabera[49] | 36/F | African American | 36 |  |  | S | MP | MP+HCQ/mycophenolate mofetil |  | no recurrences |
| Ayari, [20] | 37/F | Caucasian | 37 |  |  | S,L | MP | Pred+HCQ |  | no recurrences |
| Hsu,D[50] | 7/F | Pacific Islander | 7 | 8kg |  | / | MP+CTX,rituximab | mycophenolate mofetil |  | no recurrences |
| Lan.[51] | 18/F |  | 18 |  | 25 | S | MP+mycophenolate mofetil | MP+mycophenolate mofetil+HCQ |  | no recurrences |
| Ohri,[52] | 12/F |  | 12 |  |  | duodenum ,jejunum, | MP+IVIG | MP+CTX/mycophenolate mofetil |  | no recurrences |
| Qi.[53] | 22/F |  | 12 |  |  | / | MP+CTX | Pred+CTX |  | no recurrences |
| Wen[19] | 31/F |  | 31 |  |  | S,L | MP+CTX(-) |  | laparotomy | no recurrences |
| NAeem[54] | 36/F | Pakistani | 36 |  | 24 | / | MP+HCQ+CTX | MP+HCQ |  | no recurrences |
| Kranthi[55] | 25/F |  | 25 |  |  | stomach, duodenum, S,L | MP/Pred | Pred/mycophenolate mofetil+HCQ |  | no recurrences |

F=Female,M=Male，W=week，S=small bowel,L=large intestine,MP=Methylprednisolone，HCQ=Hydroxychloroquine, Pred=Prednisone,AZA = Azathioprine,CsA = Cyclosporine A,IVIG = Intravenous Immunoglobulin,IG = Immunoglobulin,CTX = Cyclophosphamide,5-FU = 5‑Fluorouracil

Supplementary Table2

Laboratory indices and other organ involvement in the 54 patients.

| publication | CBC | immunity index (+) | C3\C4 | urine routines | Urinary system | Other systems | Gastrointestinal symptoms |
| --- | --- | --- | --- | --- | --- | --- | --- |
| MUNYARD P et al.[23] | WBC、LY、Hb、PLT↓ | anti-dsDNA |  |  |  | Pneumococcal sepsis、 Pericardial effusion、pleural effusion | Abdominal pain,bloating,,vomiting,constipation |
| KAMIUCHI Ket al,[24] |  | ANA、anti-RNP |  |  | Hydronephrosis、Lupus cystitis |  | Abdominal pain, diarrhea, nausea, vomiting, |
| GPerlemuter et al[25] |  | ANA、anti-dsDNA、antiSM |  |  |  | The thickness of the bladder wall increases and bilateral ureters dilate | Abdominal pain, vomiting, abdominal distension, diarrhea, constipation, |
|  |  | ANA、anti-RNP |  |  |  | The thickness of the bladder wall increases and bilateral ureters dilate | Nausea, vomiting, diarrhea and abdominal pain |
|  |  | ANA |  |  |  | The thickness of the bladder wall increases and bilateral ureters dilate | Abdominal pain ,diarrhea |
| PA Het al[26] |  | ENA、anti-RNP、anti-SM | ↓ | Proteinuria (4.5 g/d) |  |  | Vomiting,abdominal pain. |
| MY Mok et al[27] |  | ANA、 anti-dsDNA、anti-Ro | ↓ | proteinuria | glomerulonephritis、glomerulonephritis | exudative right pleural effusion and ascitic fluid | abdominal pain |
|  | thrombocytopenia | ANA、 anti-dsDNA、anti-Ro | ↓ | proteinuria (1.8 g/d) | mesangial proliferative glomerulonephritis | ascitic fluid | abdominal pain,vomiting |
|  |  | ANA、 anti-dsDNA anti-Ro 、IgG ACA | ↓ | proteinuria (4.6g/d) |  | visual blurring and bilateral papilloedema、 brain multiple vasculitic lesions. |  |
|  | lymphopenia thrombocytopenia | ANA、 anti-dsDNA 、anti-Ro、 anti-RNP 、IgG ACA | ↓ |  | bilateral ureterohydronephrosis | generalised lymphadenopathy、polyarthralgia | abdominal distension, diarrhoea |
|  |  | ANA、 anti-dsDNA | ↓ |  | bilateral ureterohydronephrosis | ascitic fluid、idiopathic thrombocytopenia purpura at the age of fourteen | nausea, vomiting, abdominal pain ,abdominal distension |
|  | leucopenia、thrombocytopenia | ANA、anti-Ro、IgG and lgM ACA | ↓ | proteinuria | distended renal pelvis on the right side、focal proliferative glomerulonephritis |  | frequent vomiting (10 episodes per day) intractable to anti-emetics, abdominal pain ,diarrhea |
| J.Narvaez[28] |  | ANA、 anti-dsDNA | ↓ |  |  |  | nausea, vomiting, poor appetite,diarrhoea |
| Alexopoulou[29] |  | ANA、 anti-dsDNA 、 anti-RNP | ↓ |  | Hydroureter and hydronephrosis |  | abdominal pain, diarrhoea、vomiting |
| Mao-Yuan Chen[30] |  | ANA、 anti-dsDNA 、IgG ACL、anti-Ro | ↓ | proteinuria(0.058 g/dL) | bilateral ureterohydronephrosis. | massive ascites, enlarged mesenteric lymph nodes | Abdominal pain, nausea, vomiting,diarrhoea |
|  | Immune Thrombocytopenia | ANA 、anti-Sm、anti-RNP、anti-SSA、anti-dsDNA、IgG ACL | ↓ | proteinuria | bilateral ureterohydronephrosis with thickening of the bladder wall was shown in abdominal sonography. | hallucination and sudden blindness occurred | persistent watery diarrhoea |
|  | anaemia 、 leukopenia | ANA、anti-SSA、anti-RNP、 IgG aCL、 anti-dsDNA | ↓ | proteinuria | right ureterohydronephrosis、acute cystitis | polyarthritis | abdominal pain, nausea, vomiting,abdominal distension |
|  | Thrombocytopenia、lymphopenia、albumin | ANA、anti-SSA,、anti-dsDNA 、IgG aCL | ↓ | proteinuria | Bilateral hydronephrosis、chronic cystitis. | ascites and pleural effusion \esions in the bilateral occipital and left temporoparietal areas. | Vomiting,diarrhea,abdominal distension, anorexia, and constipation |
| Yang-YuanChen[31] |  | ANA、 anti-dsDNA、Anti-Sm/RNP, anti-Sm, anti-SSA, anti-SSB | ↓ |  | bilateral hydroureter. | massive ascites | Abdominal pain, distension |
| Maruoka [32] |  | ANA | ↓ |  | bilateral hydronephrosis, and thickened bladder wall | ascites | Abdominal pain, diarrhea, vomiting, weight loss, urinary incontinence |
| Ceccato[33] |  | ANA、RO, LA, SM、ACA | ↓ |  |  |  | Abdominal pain, diarrhea, vomiting |
|  | Anemia、lymphopenia | ANA、SM | ↓ |  | bilateral ureteral distension.and interstitial cystitis. | mild ascites | Abdominal pain, diarrhea, vomiting, weight loss, urinary incontinence |
|  |  | ANA、RO, LA | ↓ |  | bilateral ureteral distension. | mild ascites | abdominal discomfort, distension, obstipation |
|  | leucopenia, lymphopenia、albumin↓ | ANA、RNP、anti-dsDNA |  |  |  | ascites and right pleural effusion. | postprandial fullness , vomiting,abdominal bloating, dysphagia with solid foods,abdominal pain with cramps. |
| Frederick D[34] |  |  |  | proteinuria | acute renal insufficiency、bilateral hydroureters and hydronephrosis、lupus cystitis | intraand extra-hepatic biliary tree dilatation、ascites, arthralgias, |  |
| Leonardi,G[35] | anemia、leukopenia |  | - |  |  |  |  |
| Ghannouchi Jaafoura[36] |  | ANA、 anti-dsDNA 、APL |  | Proteinuria | bilateral ureterophageal dilatation |  | Diarrhea,vomiting, abdominal pain ,distension vomiting,abdominal pain ,distension |
| kim[37] |  | ANA 、anti-Sm、anti-RNP |  | Proteinuria |  |  | paroxysmal crampy upper abdominal pain, nausea, vomiting, and constipation with moderate fever. - abdominal distension with passage of loose stools |
| zhang[38] | Thrombocytopenia、Hypokalemia | ANA、ds-DNA、u1RNP、SSA and SSB、IgG ACA | ↓ | Candida albicans 、proteinuria | bilateral ureterohydronephrosis with distended pelvis (Figure 1D) and ureter | Peritonitis | recurrent vomiting, diarrhea |
|  | Plasma albumin ↓ 、eosinophils↑ | anti-ANA 、 Sm, u1RNP、 SSA、SSB |  | hematuria、 proteinuria | bilateral ureterohydronephrosis | ascites and pleural effusion. peritonitis | severe colicky abdominal pain, recurrent vomiting and constipation |
| Chen[14] |  | ANA、 | ↓ |  | Hydronephrosis | arthralgia、ascitic fluid,,right hydronephrosis and hydroureter (a), intra and extrahepatic biliary tree dilatation and megacholedochus | abdominal discomfort and distension,recurrent nausea ,vomiting, profuse diarrhoea |
| Kansal,A[39] | Hypokalemia | ANA、 anti-dsDNA 、 anti-RO | ↓ |  | Again dilated intrahepatic biliary radicles were seen with presence of bilateral hydroureteronephrosis | ascites. | abdominal pain、pleural effusion. |
| Khairullah [40] | Anaemia、thrombocytopaenia、AIHA | anti-SSA and anti-SSB. | ↓ |  | bilateral ureterohydronephrosis | ascites.enlarged mesenteric lymph nodes | abdominal pain, vomiting ,oral intake intolerance. |
| Garcia Lopez[41] | hyperazotemia, hemolytic anemia | ANA, anti-DNA, anti-SM and anti-Ro | ↓ | proteinuria (up to 21 g/24 h)、 E. coli | dilatation of the pyelocalyceal systems, ureterohydronephrosis and emphysematous pyelonephritis | central nervous system activity after resonance angiography, lumbar puncture and cerebral perfusion scan. | Diarrhea, abdominal pain ,vomiting |
|  | leucopenia, lymphopenia, hemolytic anemia | ANA and anti-DNA、Anti-Ro | ↓ | proteinuria (up to 10.76 g/24 h)、 E. coli. |  | ascites and splenomegaly | vomiting |
|  | leukopenia 、lymphopenia, | anti-dsDNA, ANA 、Anti-Ro | ↓ | proteinuria (10 g/24 h) 、E. coli --anuria | acute renal failure、ureterohydronephrosis | acute pulmonary edema | nausea、vomiting-abdominal distension. |
| wang[42] | hypoproteinemia、hypokalemia | anti-dsDNA, ANA | ↓ |  |  |  | abdominal pain, nausea, vomiting and constipation |
| oh[1] | Hypokalemia | ANA、Anti-Ro 、antiLa 、anti-Ro52 |  |  |  |  | abdominal pain,abdominal distension, constipation, |
| Ji,C[43] | Hypokalemia | ANA\anti-ribo\ anti-Smith\ anti Sjogren's syndrome A \ anti-Sjogren's syndrome B | ↓ |  |  | ascites\ peritoneal fluid 、bilateral hydrothorax | recurrent abdominal pain, vomiting, diarrhoea |
| Yang,C[44] | leukopenia | ANA (titer 1:2,560), anti-dsDNA (titer>900), anti-Sm (+), | ↓ |  | ureterohydronephrosis and urinary  retention | joint disease, and pleural eff usions. | abdominal distension, colicky pain ,loose stool |
| Shirleley[45] |  |  | ↓ |  | bilateral tortuous dilated uretero-hydronephrosis \neurogenic bladder with vesicoureteric reflux. | ascites. | abdominal pain ,distension,nausea, vomiting, |
|  |  |  |  |  | mild hydronephrosis and hydroureter bilaterally/ thickened urinary bladder wall | ascites. | abdominal pain, distension, and emesis. |
| wang[46] | hypoalbuminemia | ANA、SSA-52、SSA-60 |  | proteinuria | hydronephrosis, dilation of both renal pelvis、active focal lupus nephritis with membranous lupus nephritis | effusion in thoracic cavity, abdominal cavity and pelvic cavity | recurrent abdominal pain ,vomiting |
| Adler[47] | leukocytosis | ANA、RO、anti-RNP | ↓ | Escherichia coli | bilateral hydronephrosis.membranous lupus glomerulopathy (class V) with focal mild mesangial proliferation. | ascites | abdominal distension, diarrhea, nausea,vomiting |
| Zhang,j[48] |  | ANA,anti-dsDNA antibody (+), anti-histone antibody (+), and anti-nucleosome antibody (+). |  | Proteinuria、haematuria, |  | bilateral lung infection, bilateral pleural effusion, pericardial effusion, splenomegaly, enlarged retroperitoneal lymph nodes, and pelvic effusion | diarrhea |
| Nyabera[49] |  | ANA, anti-DNA, anti-SM、aCL | ↓ |  | bilateral hydroureteronephrosis | and moderate intra-abdominal ascites | abdominal pain, fatigue, constipation, ageusia, anorexia,nonbloody, nonbilious emesis. |
| Ayari, [20] | Anemia、leucopenia、lymphopenia、hypokalaemia | ANA, anti-DNA, SSA\SSB |  |  |  | bilateral pleural, pelvic, abdominal effusion | abdominal pain, abdominal distension, vomiting |
| Hsu,D[50] | Hypoalbuminemia、anemia、leukopenia 、lymphopenia | ANA 、anti-Sm、anti-RNP | ↓ |  |  | a small pericardial effusion on echocardiogram, | abdominal pain with distension, bilious vomiting, constipation |
| Lan.[51] | leukopenia | anti-RNP、SSA、SSB、P-ANCA、ANA、anti-dsDNA | ↓ | proteinuria |  |  |  |
| Ohri,[52] |  | ANA, anti-DNA, | ↓ | proteinuria | membranous lupus nephritis class V | bilateral pleural effusion\Ascitic | stopped defecating, and this was accompanied by abdominal distension, intermittent nausea and retching. |
| Qi.[53] |  | ssA |  |  | bilateral ureterohydronephrosis |  | recurrent episodes of diarrhea, vomiting, generalized abdominal pain,loose stools 、 constipation |
| Wen[19] |  | ANA | ↓ | proteinuria |  | dilated intrahepatic and extrahepatic bile ducts and dilated pancreatic ducts; significant enlargement of the gallbladder; bilateral dilatation of the renal pelvis, calyces and ureter; | diffuse abdominal pain, vomiting and abdominal distension |
| NAeem[54] | hemolytic anemia, hypokalemia | anti-nucleosome antibodies (8 U/mL), equivocal anti-dsDNA antibodies (7 U/mL) and anti-Sm antibodies (7 U/mL), and positive anti-Ro antibodies (100 U/mL) and anti-La antibodies (53 U/mL). | ↓ | proteinuria | bilateral hydronephroureter, and thick-walled urinary bladder. |  | Abdominal pain, bloating,,vomiting and constipation |
| Kranthi[55] | hypokalaemia | ANA and anti-DNA、Anti-Ro-52、anti-sm | ↓ | Proteinuria、microhaematuria, |  |  | Abdominal pain, diarrhea, nausea, vomiting, |

WBC = White Blood Cell,LY = Lymphocyte,Hb = Hemoglobin,PLT = Platelet·
